# Hypothesis: Caco‐2 cell rotational 3D mechanogenomic turing patterns have clinical implications to colon crypts

**DOI:** 10.1111/jcmm.13853

**Published:** 2018-09-25

**Authors:** Gen Zheng, Alexandr A. Kalinin, Ivo D. Dinov, Walter Meixner, Shengtao Zhu, John W. Wiley

**Affiliations:** ^1^ Division of Gastroenterology Department of Internal Medicine University of Michigan Medical School Ann Arbor Michigan; ^2^ Department of Computational Medicine and Bioinformatics University of Michigan Medical School Ann Arbor Michigan; ^3^ Statistics Online Computational Resource (SOCR) University of Michigan School of Nursing Ann Arbor Michigan; ^4^ Michigan Institute for Data Science (MIDAS) University of Michigan Ann Arbor Michigan; ^5^ Department of Digestive Diseases Beijing Friendship Hospital Capital Medical University Beijing China; ^6^ National Center for Clinical Medical Research of Digestive Diseases Beijing China

**Keywords:** 4D nucleome, colon crypt, colorectal cancer, functional bowel disorders, glucocorticoid, HES1, mechanobiology, Notch, tight junction, turing pattern

## Abstract

Colon crypts are recognized as a mechanical and biochemical Turing patterning model. Colon epithelial Caco‐2 cell monolayer demonstrated 2D Turing patterns via force analysis of apical tight junction live cell imaging which illuminated actomyosin meshwork linking the actomyosin network of individual cells. Actomyosin forces act in a mechanobiological manner that alters cell/nucleus/tissue morphology. We observed the rotational motion of the nucleus in Caco‐2 cells that appears to be driven by actomyosin during the formation of a differentiated confluent epithelium. Single‐ to multi‐cell ring/torus‐shaped genomes were observed prior to complex fractal Turing patterns extending from a rotating torus centre in a spiral pattern consistent with a gene morphogen motif. These features may contribute to the well‐described differentiation from stem cells at the crypt base to the luminal colon epithelium along the crypt axis. This observation may be useful to study the role of mechanogenomic processes and the underlying molecular mechanisms as determinants of cellular and tissue architecture in space and time, which is the focal point of the 4D nucleome initiative. Mathematical and bioengineer modelling of gene circuits and cell shapes may provide a powerful algorithm that will contribute to future precision medicine relevant to a number of common medical disorders.

## 1 GENERAL HYPOTHESIS

Alan Turing made the initial proposal that it is feasible to mathematically model specific patterns observed in multicell systems in his 1952 seminal paper “The Chemical Basis of Morphogenesis”.^1^ He mentioned that “The purpose of this paper is to discuss a possible mechanism by which the genes of a zygote may determine the anatomical structure of the resulting organism.”, he also suggested that “The genes themselves may also be considered to be morphogens.”.^1^ Turing was limited by the lack of availability of appropriate data: “There are probably many biological examples of this metabolic oscillation, but no really satisfactory one is known to the author.”.^1^ The NIH launched the 4D Nucleome program to understand the principles behind the 3D organization of the nucleus in space and time (the 4th dimension), the role nuclear organization plays in gene expression and cellular function, and how changes in the nuclear organization affect normal development as well as various diseases. Following Turing's inspiration, 3D‐FISH has been employed to track movements of oscillatory genes in synchronized cells to generate data required for producing 4D Nucelome algorithms of the genome.^2^, ^3^ Smale and Rajapakse expanded the Turing “gene morphogen” proposal in publications that updated mathematical models able to handle extra dimensions in addition to 2D Turing pattering and proposed implications relevant to the 4D Nucleome initiative.^2^, ^4^, ^5^ Colon crypts are viewed as a 2D Turing patterning multicell system based on cell‐cell junctional forces or transcription factors HES1 and MATH1 mathematical analysis.^6^, ^7^ The neuroectoderm Turing patterning Delta‐Notch lateral inhibition mechanism has been mathematically verified in colon crypts.^6^ Relevant to this article, overexpression of colon epithelial cell tight junction protein CLDN1 (claudin 1) increases Notch activity measured by HES1 and MATH1 expression.^8^ Notch signalling target HES1 transcription can be triggered by mechanical forces that pull cells apart which activate Notch cleavage.^9^ CLDN1 holds cells together which counteracts the pulling force. These features provide a potential linkage between oscillatory actomyosin forces and oscillatory HES1 expression.^10^, ^11^ Gene regulatory circuits containing cell and developmental stage‐specific transcription factors forming “a two‐gene network with two repressors” that bind each other's promoters have been proposed as “hardwiring” required for stable equilibrium of cell types in “Mathematics of the Genome” involving “n‐Dimensional Dynamical Systems”.^5^ We observed potential “toggle gene circuits” as proposed involving the transcription factors HES1 and NR3C1 (glucocorticoid receptor, GR) that regulate CLDN1 along the human colon crypt axis, and *in vitro* Caco‐2 cell differentiation.^5^, ^12^, ^13^, ^14^ We propose that modelling the 4D Nucleome dynamics, 4D mRNA distribution and actomyosin forces that regulate tight junction protein expression and function will predict the self‐organizing of epithelial cells in a cell type‐, developmental stage‐specific manner. This information will be useful in generating a precise mathematical model of human colon crypts, which could be employed as a powerful algorithm to help design precision medicine approaches for targeted, disease‐specific treatments in a variety of medical ailments, including functional bowel disorders (FBD) and colorectal cancer (CRC).^5^, ^12^, ^14^, ^15^, ^16^

**Figure 1:**
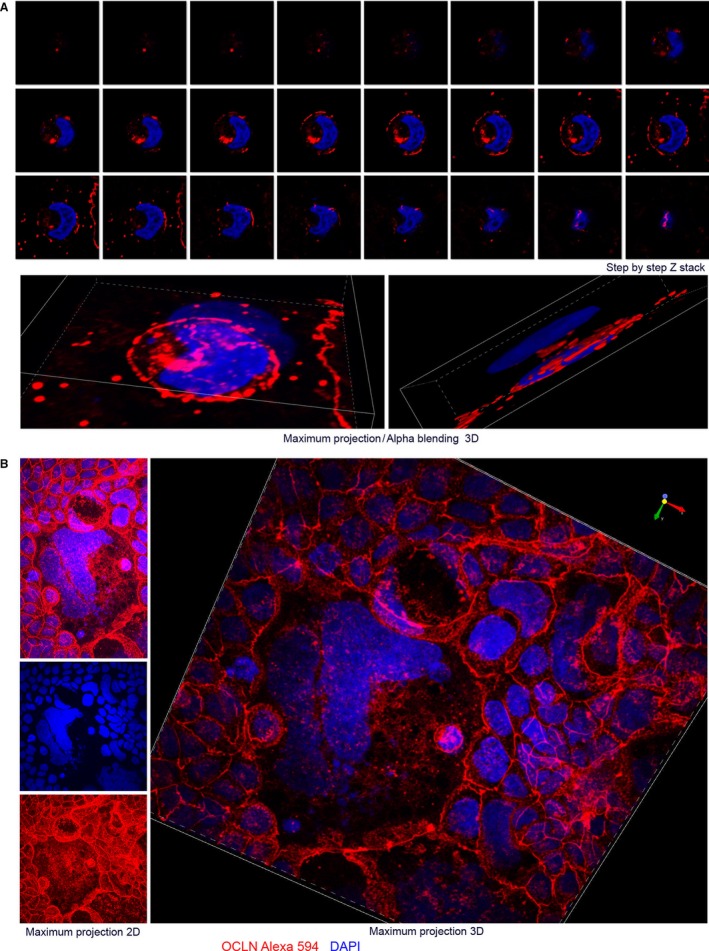
Fractal rotational patterns of tight junctions and nucleus DNA. Caco‐2 BBe cells on day 6 cover clips are labelled with OCLN protein and DNA. Yin‐yang (YY) shapes, small YY (A) in big YY (B). YY shape may correlate with rotational motion and symmetry breaking.

To generate “proof of concept” data, we tracked the formation of a coordinated epithelial cell sheet during Caco‐2 cell differentiation on a smooth, flat and hard glass surface that recapitulates known gene expression patterns that occur along the colon crypt axis. Detailed in‐depth description and discussion of the rotational 3D mechanogenomic Turing patterns observed during differentiation are included in the supplementary and online material (Figure 1 and S1) (http://www.socr.umich.edu/projects/3d-cell-morphometry/data.html).^17^, ^18^, ^19^, ^20^

## 2 SOME POTENTIAL APPLICATIONS OF TURING PATTERN ANALYSIS IN GASTROINTESTINAL DISORDERS

### 2.1 Functional bowel disorders (FBD) and colorectal cancer

Functional bowel disorders including irritable bowel syndrome (IBS) represent dysfunction in the bidirectional brain‐gut axis, intestinal barrier integrity and interactions with the microbiota and dietary factors.^21^ Clinical colonoscopy biopsies harvested from diarrhoea‐predominant IBS (IBS‐D) patients demonstrated decreased CLDN1 levels, while CLDN1 was increased in constipation‐predominant IBS (IBS‐C) patients.^22^ The CLDN1 promoter is under the dual reciprocal regulation by HES1 and NR3C1 in Caco‐2 cells and a validated chronic, intermittent water avoidance (WA) stress rat model of stress‐induced enhanced abdominal pain that mimics several clinical features observed in IBS‐D patients.^12^ We observed down‐regulation of both HES1 and NR3C1 via a glucocorticoid negative feedback pathway in WA‐stressed rat colon crypts, and similar trends were observed in the hippocampus in a validated restraint‐stress mouse model demonstrating anxiety and depression‐like behaviours.^12^, ^23^ Deletion of the Notch signalling ligand Delta‐like 2 (DLK2) increased anxiety and depressive‐like behaviours and altered the vulnerability to restraint stress, and reversed stress‐induced down‐regulation of NR3C1 and HES1.^23^ HES1 is responsible for maintaining gut homeostasis via preventing microbial dysbiosis in the mouse, and HES1‐knockout altered colon crypt morphology.^24^ The probiotic combination of *Lactobacillus helveticus* and *Bifidobacterium longum* helped reverse WA‐stress‐induced changes in the mouse hypothalamic‐pituitary‐adrenal axis and WA stress‐induced visceral hyperalgesia by blocking decrease of NR3C1 in the hypothalamus, hippocampus and prefrontal cortex.^25^ These reports support the potential of HES1‐CLDN1 and NR3C1 acting as equilibrium maintaining gene circuits consists of three genes that regulate each other in a cyclical manner and their potential roles in homeostasis of the Microbiota‐Gut‐Brain Axis. Future advances in personalized probiotics based, in part, on 4D Nucleome algorithms represent a potentially promising therapeutic area.^21^, ^25^

**Figure 2:**
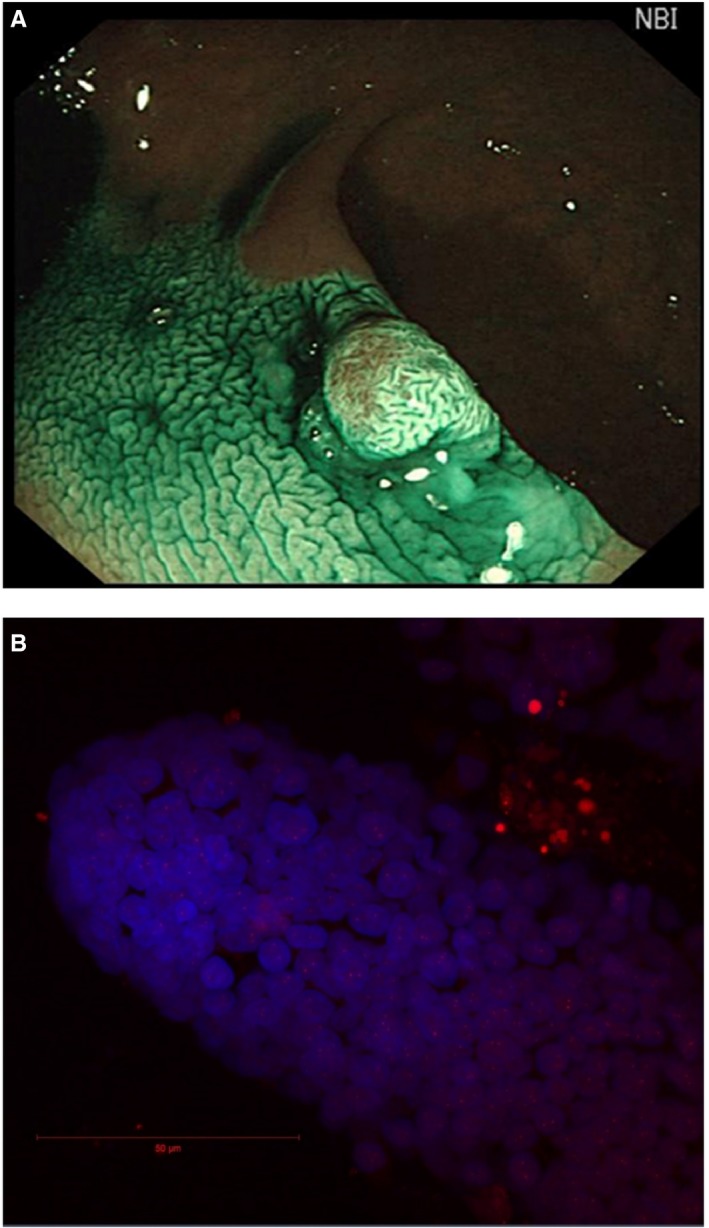
Human colon crypts could be isolated form Turing patterning epithelium for 4D‐nucelome analysis. A, An example of colonoscopy image containing 2D/3D Turing patterns.^30^, ^31^ B, An example of BAC‐FISH analysis of 3D human colon crypts isolated from clinical biopsy

Colorectal cancer is the second most common cancer in women and the third most common in men, CLDN1 is recognized as a potential biomarker.^26^ Overexpression of CLDN1 induced elevated levels of Wnt and Notch signalling, promoted colon tumorigenesis in mice, and altered goblet cell differentiation, which conforms to 2D colon crypt Delta‐Notch lateral inhibition Turing patterning.^6^, ^8^ Turing models of metabolism in colon cancer link Wnt signalling and gene circuits. We propose that these gene circuits represent potentially promising novel colon cancer targets to model mathematically.^12^, ^27^ A preclinical confocal colonoscopy study demonstrated that a CLDN1‐binding peptide can visualize overexpressed CLDN1 in colonic adenomas *in vivo*, and actomyosin meshwork measurements have been used to demonstrate morphogenesis. Therefore, it is feasible to combine these techniques to assess tight junction geometric patterns potentially useful to monitor the transformation of normal to abnormal (e.g. cancer) actomyosin meshwork pattern(s).^3^, ^28^, ^29^ Human colon epithelium demonstrates various patterns during colonoscopy that correlate with different physiological and pathological conditions, which can be recognized using recently developed artificial intelligence algorithms.^16^, ^29^ 2D/3D patterns consistent with Turing's hypothesis appear to be present in our clinical data set (Figure 2A).^30^, ^31^ We propose that these distinctive colon mucosa patterns support the presence of morphogenetic feedback mechanisms which are hypothesized to regulate the topological architecture of tissues.^3^, ^32^ Integrating our knowledge about gene circuits and tissue topology mathematically may help to guide the development of novel interventions targeting the glucocorticoid/Notch/Wnt pathways.^8^, ^13^, ^27^, ^33^ Dynamic 4D Nucleome algorithms may provide a more powerful predictive model of colon cancer with the applications of machine learning methods.^16^

### 2.2 Analysis and bioengineering of human colon crypts

In addition to robust‐omics analysis including RNA‐seq, emerging 4D Nucleome algorithms demonstrate the potential application to pharmacogenomics.^34^ Clinical colonoscopy biopsies can be used to isolate intact crypts with the structure similar to gastrulation as mentioned by Turing.^1^ Given biopsy samples, clumps of normal/cancer cells can be fixed and imaged using the same confocal microscopy methods used for the coverslip. Then, the same protocol for automated cell and subnuclear structure labelling can be applied to generate solid volumetric masks of nuclear components. Our pipeline workflow protocol may need to be tailored to each new imaging data format; however, the overall design of the protocol is not expected to drastically change.^17^, ^18^, ^19^, ^20^, ^35^ Application of BAC‐FISH methods to study colon crypts is feasible in our pilot study (Figure 2B). Correlating morphogenesis with oscillatory gene movement and expression including clarification of the biophysical mechanisms that determine DNA‐histone interactions and their regulation by cooperative transcription factors will be required to generate convincing proof of Turing's “gene morphogenesis hypothesis” in primary cells and tissue models.^1^, ^2^, ^3^, ^14^, ^36^

We anticipate that application of *in vitro* mechanobiology culture methods to isolated human colon crypts and stem cells will replace the Caco‐2 cell model in testing the gene morphogen hypothesis with programmable morphogenesis parameters.^37^, ^38^ Readily available biopsy specimens demonstrating distinct epithelial lumen surface Turing patterns can be used to isolate crypts to study stem cells present at the crypt base from healthy normal and abnormal epithelium using *in vitro* organoid preparations (Figure 2A).^39^ We anticipate the programmable “gut‐on‐a‐chip” methodology will demonstrate epithelium morphology similar to the colon lumen surface. This system can be used to study the topology of colon epithelium in response to morphogens including FGF2/glucocorticoids/microbiome, etc.^25^, ^40^, ^41^, ^42^ High‐throughput “gut‐on‐a‐chip” array methods allow automated experimental designs incorporating various chemical, medicinal and mechanical morphogen conditions. These approaches will generate large datasets requiring the concurrent development of machine learning algorithms to expedite analysis, and potentially confirmation of the “gene morphogen hypothesis”.^16^, ^43^, ^44^

### 2.3 Mathematical/computational problems unresolved by Alan Turing's Model

Testing the hypothesis presented in this communication will require advanced analytical techniques to generate the relevant information regarding the determinants of cell and tissue morphology patterns under physiologic and pathophysiologic conditions. Prior computational models suggested considering the nucleus as a “cellular decision‐making unit” that is a pivotal component in “cellular decision‐making networks”.^45^ Computational modelling of cellular “decisions” in response to multiple biochemical and biophysical cues will require a level of mathematics capable of “handling” multiple dimensions.^45^ Advanced mathematical methods including chaos theory and fractal geometry, in addition to the “relatively elementary” linear models and differential equations used by Alan Turing will be required to explore “The Secret Life of Chaos” of gut homeostasis.^1^, ^4^, ^5^, ^46^ Fractal geometry was developed to understand self‐similar structure at multiple scales. It provides the powerful strategies for analysing self‐similar shapes using efficient computational algorithms. Growth spiral (Logarithmic Spiral) is a self‐similar spiral curve which often appears in nature, it is frequently used to demonstrate fractal geometry which was known as known as expanding symmetry or evolving symmetry. Self‐similar spirals with various parameters are used to illustrate bifurcation of two‐gene networks responsible for cellular decision‐making and “tissue homeostasis”.^4^, ^5^ Self‐similar genome (DNA) and tight junction shapes are observed in our preliminary study, rotational motion of the nucleus, HES1 mRNA/protein distribution, tight junction shapes labelled with occludin antibody seems contributed to those shapes (Figures 1, S1‐S5 and S9). Correlating visionary mathematical idealization and real subjects is very intriguing and challenging, we hope our observation and thoughts could benefit future studies.

## CONFLICT OF INTERESTS

The authors declare that declare that they have no significant competing financial, professional or personal interests that might have influenced the performance or presentation of the work described in this manuscript.

## AUTHORS’ CONTRIBUTIONS

G.Z. conceived the experiments shown in Figures 1 and 2B. S.Z. acquired image shown in Figure 2A. W.M. acquired image shown in Figure 2B. A.K., I.D.,W.M., S.Z. and J.W. participated discussion and editing. All authors reviewed the manuscript.
