# Supplementary material for: Hypothesis: Caco‐2 cell rotational 3D mechanogenomic turing patterns have clinical implications to colon crypts
Source: J Cell Mol Med. 2018 Sep 25;22(12):6380–5. doi: 10.1111/jcmm.13853 (PMC6237597; doi:10.1111/jcmm.13853)
Supplement: Supplementary file 1 [file JCMM-22-6380-s001.docx]

### Supplementary material:

### Observation of Caco-2 rotational 3D mechanogenomic Turing patterns during Caco-2 differentiation

### Abstract

Colon crypts are recognized as a mechanical and biochemical Turing patterning model. Colon epithelial Caco-2 cell monolayer demonstrated 2D Turing patterns via force analysis of apical tight junction live cell imaging which illuminated actomyosin meshwork linking the actomyosin network of individual cells. Actomyosin forces act in a mechanobiological manner that alters cell/nucleus/tissue morphology. We observed the rotational motion of the nucleus in Caco-2 cells that appears to be driven by actomyosin during the formation of a differentiated confluent epithelium. Single- to multi-cell ring/torus-shaped genomes were observed prior to complex fractal Turing patterns extending from a rotating torus centre in a spiral pattern consistent with a gene morphogen motif. These features may contribute to the well-described differentiation from stem cells at the crypt base to the luminal colon epithelium along the crypt axis. This observation may be useful to study the role of mechanogenomic processes and the underlying molecular mechanisms as determinants of cellular and tissue architecture in space and time, which is the focal point of the 4D nucleome initiative. Mathematical and bioengineer modelling of gene circuits and cell shapes may provide a powerful algorithm that will contribute to future precision medicine relevant to a number of common medical disorders.

**Background information**

We recently developed an automated 3D cell morphological classification allowing quantitative morphometry analytics of multiple geometric features, which are not accessible by traditional analysis of counting and signal intensity quantification [1]. Our shape analysis of nuclei and nucleoli of proliferating/non-proliferating fibroblast cells and epithelial/mesenchymal human prostate cancer (PC3) cells found morphology features including eccentricity and surface area to be useful predictors for these cell types. Symmetric and asymmetric cell divisions are the topological basis for the transformation from a single cell (a fertilized egg/a stem cell) to a multi-cell system (an embryo/a piece of tissue). Our morphological classification protocol can potentially be applied to discriminating non-dividing cells/dividing cells, as well as symmetrically dividing cells/asymmetrically dividing cells. Machine learning classifiers in our protocol can use topological characteristics describing patterned cells/nuclei and are directly applicable to quantifying the 4D nucleome in intact tissues. Analysis of actomyosin meshwork during embryo gastrulation (which initiates asymmetric cell division and epithelial–mesenchymal transition) also used eccentricity and surface area as “a small number of parameters” to describe the complex behaviour of the embryo tissue [2]. To help generate “proof of concept” data, in this study we chose a commonly used human colon epithelial cell model, Caco-2 BBe, tracked the formation of a coordinated epithelial cell sheet during differentiation of increased cells on a limited smooth, flat and hard glass surface. These results demonstrate the ability to model the shape challenges of the 4D nucleome using morphometric analysis, which may be applied to coordinating multi-cell systems [3,4]. Computational models with the goal to generate theoretical morphogenesis frameworks integrating the knowledge of mechanical, cellular and gene-regulatory levels may benefit from these observations [4-7].

**Observation of Turing’s 4 types of changes**

The emerging mechanobiology examines the role of physical and mechanical forces in the control of cell development and disease, in addition to chemicals and genes [8]. A major goal of the 4D nucleome project is to understand the subcellular details of this process compared to physical systems governed by Newton’s laws of motion [9]. The initial mathematical analysis of the multi-cell system using the digital computer based on Newton’s laws of motion proposed 4 types of changes involving the mechanical and the chemical parts:

“(i) The changes of position and velocity as given by Newton's laws of motion.

(ii) The stresses as given by the elasticities and motions, also taking into account the osmotic pressures as given from the chemical data.

(iii) The chemical reactions.

(iv) The diffusion of the chemical substances. The region in which this diffusion is possible is given from the mechanical data.”

These processes are articulated in Alan Turing’s 1952 paper [10]. Based on these assumptions, Turing predicted specific shapes would be observed in biological systems at the cellular and tissue levels, e.g., “ring of cells” in 2D & gastrulation in 3D [2,7,11].

Rotational nucleus motion driven by actomyosin is observed within single cells [12]. Epithelial cells demonstrated rotational motion capabilities in 2 cell model, which was correlated with polarities [13] (Fig. S1-S3). The rotational motion during 3D morphogenesis from a single cell to a 3D epithelium sphere was also detected [14]. The sphere surface tight junction curves look like rubber bands demonstrating different eccentricity values (circle/ellipse/parabola/hyperbola) which are assumed to be generated by rotational motions and might be used as basic elements for nucleus and cell morphology analysis (Fig. S4-S6) [1,2,13]. Coordinated cell behavior and shape characterization have been observed in 1 to 2 models, and multiple cells. However, new higher-resolution 3D/4D imaging and post-imaging analysis will be required to precisely track the coordinated 4D dynamics of patterned cells and nuclei.

Mechanical forces generated by the actomyosin network can force the cells and nuclei into different shapes [15,16]. The forces from actomyosin network can be transduced to the genome via LINC (linker of nucleoskeleton and cytoskeleton) complex to the genome and induce epigenetic, and transcriptional changes [8,17-19]. Human colon epithelial Caco-2 cells during the early stage of differentiation (2-4 days) demonstrated quantifiable changes of nuclear and nucleolar shape morphology detectable by our shape classification protocol [1,20]. Epigenetic modification H3K9me3 is sensitive to the mechanical forces, robust upregulation of H3K9me3 by mechanical force was observed within 1 min, and culture matrix stiffness can mediate H3K9 methylation [21,22]. We observed the signature rotational pattern of H3K9me3 signals within the Caco-2 nuclei coordinating with each other, where the signals seemed to be correlated with mechanical pressure spots (Fig. S7). Our shape classification protocol assesses the morphometry of nuclei and nucleoli that can be used as quantitative signature vectors for analysing the epigenetic modification shape variations between the cells, which are difficult to interpret using traditional methods based on homogenizing the cells (e.g. ChIP-seq) [1].

Mechanical crosstalk between cells via apical tight junctions of epithelial cells membrane mediate the functional signal transduction and pathogenesis [23-25]. Caco-2 is a frequently used cell model for epithelial mechanobiology/morphogenesis. It can form: (1) 2D epithelial membrane; (2) 3D epithelial sphere/cyst/tube; or (3) dynamic membrane with villin structures close to *in vivo* gut epithelium with different mechanobiology culture conditions [14,26,27]. The 2D culture of this model has been used for decades to study gut barrier function and paracellular permeability dependent on the expression and function of epithelial cell tight junctions [28]. These were also mathematically validated as a mechanobiology model of 2D Turing patterning. Self-organizing actomyosin meshwork was observed and simulated to drive the oscillatory patterning the cells at epithelial apical tight junctions. The actomyosin forces demonstrated a circular wave pattern [29]. Actomyosin oscillation is important in tissue-level and cell-cell coordination, including the epithelium [30]. The 3D culture of a single Caco-2 cell to a polarized epithelial sphere is also used to study the molecular mechanism of epithelial morphogenesis involving symmetric and asymmetric divisions via various interventions [14,31-33]. The actomyosin network is responsible for the morphology of the 3D sphere/cyst formation of Caco-2 cells [26]. Rotational motion during the 3D morphogenesis may correlate with the formation of the Turing-proposed “ring of cells”/dome observed on flat glass coverclips (Fig. S8-S9) [10,14]. The formation of “rings of cells” or “domes” is also observed in functional enterocyte epithelium differentiated from human induced pluripotent stem (iPS) cells, which could be used to replace the Caco-2 cell model. In wound healing morphogen FGF2 treatment, the latter inhibits differentiation and prevents the formation of “ring of cells” in Caco-2 cells [7,11,34]. Knockdown of the [extracellular matrix](https://en.wikipedia.org/wiki/Extracellular_matrix) protein which stimulates differentiation in Caco-2 cells delayed the “ring of cells” formation [35]. Apical tight junctions are also involved in maintaining the polarity and the actomyosin network controlled the “dividing angle” of the epithelial cells in 3D morphogenesis [36]. Tight junction containing cellular structure midbodies are recognized as the organelle regulating symmetric and asymmetric cell division which determine the cell fate [37] (Fig. S6). Rotational motion-generated “Yin-yang (YY)” shape has been proposed to correlate with the breaking of mammalian cell symmetry which is “essential for cell movement, polarity, and developmental patterning” [13,38]. We observed large fractal YY shaped cells in our study (Fig.1 and Supplementary Movie.S1).
Mechanical forces can trigger the biochemical Notch signaling pathway which is considered a sensor of mechanobiology signals. These mechanical forces pull cells apart leading to proteolytic activation of Notch while tight junctions holding cells together apparently counteract against the pull [8,39,40]. This process contributes to Notch signaling pathway’s ability to integrate multiple micro-environmental signals and sense cell-cell interaction, trigging the downstream Notch signaling target HES1 transcription [41,42]. Thus, cell shapes can affect the Notch signaling triggered events [43]. Key transcription factor “master” regulators are considered important 4D nucelome modulators [5,7,9]. NR3C1 and HES1 can bind their own promoters for dynamic autoregulation, they can also repress each other’s transcription via binding to the promoters [44-46]. This special “hard-wiring” matches with “a two-gene network with two repressors” model supports Turing’s patterns [4,6]. We found that these motifs displayed reciprocal expression during Caco-2 cell differentiation and along the basal-apical axis in human colon crypts, which could be simulated by calculating forces between the cells via cell shapes [5,47]. This pattern conforms to “a stable equilibrium as a cell type” though differentiation in a mathematical model of the genome which includes the toggle gene circuits (from high averaged surface area HES1+/NR3C1- PRC2 and H3K27me3+ to low averaged surface area HES1-/NR3C1+ PRC2 and H3K27me3-) [6,48]. HES1 is an extensively studied and mathematically simulated oscillatory transcription factor, that is also involved in 2D epithelium Turing patterning [49,50] (Fig. S1, S10). Recent mathematical models confirm that the HES1 promoter is the crosstalk hub in Notch-Wnt interactions of the intestinal crypts, and our recent study also suggests that the glucocorticoid (GC) signal can affect HES1 expression via HES1 promoter negative glucocorticoid response element (GRE) [5,51,52]. We observed down-regulation of HES1 by psychological stress-mediated elevation in corticosterone. HES1 down-regulation resulted increased goblet cell population, altered colon crypts morphology and impaired gut homeostasis which conform to the mathematical model [5,51,53]. These features indicate that the HES1 promoter is also a potential crosstalk hub of Notch-GC interactions in colon crypts [5,52]. The levels of these opposite transcription factors correlated with tight junction protein levels. We observed robust down-regulation of gene oscillator HES1 and robust up-regulation of tight junction protein CLDN1 correlated with NR3C1 upregulation during Caco-2 cell differentiation, *in vitro*. These features appear to correlate with the morphogenesis of intestinal crypts structure [7]. We hypothesize that there is a hardwired antagonism between tight junction CLDN1 gene and HES1 gene coded close on chromosome 3 in opposite directions too. Their promoter positive/negative GREs and HES1 binding N-boxes provided regulatory elements required for dynamic regulation [5]. “Hardwiring” of HES1-CLDN1 and NR3C1 may meet the proposed criteria of a repressilator in a mathematical model of the genome [4,6]. Notch’s mechanical feature and the proposed transcription factor/promoter hardwiring support potential hardwired linkage between oscillatory actomyosin force and oscillatory gene transcription in the scenario of morphogenesis [40,42,54,55]. We propose that HES1-CLDN1 and NR3C1 genes could be candidates to test the gene morphogen hypothesis and investigate the 4D nucleome dynamics during differentiation and morphogenesis [5,6,56]. The microtubule-dependent global mRNA polarization was recently verified in the intestinal epithelium [57]. This is consistent with our observation of polarized HES1 mRNA distribution (Fig. S1 & S10). Asymmetric distribution of HES1 is observed with asymmetric H3K9me2 histone modification during asymmetric cell division. Therefore, they may be used as the markers in identifying proliferating, differentiating and apoptosis cells along the colon crypt axis [58] (Fig. S1, S7, S10, S11). We suggest that these markers may be useful in the topological analysis of patterning in multi-cell systems.

### Methods

**Caco-2 BBe cell culture**

Caco-2 BBe cells are kindly provided by Prof. David E. Smith, Department of Pharmaceutical Sciences, and the University of Michigan. Cells were cultured with DMEM suppled with 0.01 mg/ml human transferrin; 10 % FBS in humidified 37 °C incubator with 5 % CO_2_. Cells were seeded at 50-60 % density, the medium was replaced every 2 days. These methods are expanded versions of descriptions in our related work [5].

**Fixation**

ZEISS 0.17 mm coverclips was cleaned with cleaning solution (50% ddH_2_O, 25% ammonia and 25% Isopropanol) with 5 min agitation and washed with ddH_2_O 5 times 3 min agitation. Then the autoclaved and dried coverclips were put into non-coated 8 well plates before seeding the Caco-2 BBe cells. Fixation followed these steps: 1. 1× PBS wash twice; 2. 4% PFA/1× PBS for 10 min at room temperature; 3. 0.5% Triton-X100 in 1× PBS for 15 min; 4. 20% glycerol in 1× PBS treatment for at least 30 min; 5. 3 times repeated freeze/thaw in liquid nitrogen followed by soaking in 20% glycerol/PBS with a carbon tipped reverse tweezer; 6. 1 time wash in 1× PBS; 7. Incubate in 0.1 M HCl for 5 min; 8. Rinse in 2× SSC 9. Incubate in 50% formamide/2×SSC (at least 30mins at RT). Samples in 50% formamide/2×SSC kept in dark at 4 °C could be used 1 month after fixation. For RNA FISH, RNase free ddH_2_O/ 10×PBS/ 20×SSC were used. These methods are expanded versions of descriptions in a related work [59].

**Immunofluorescence (IF)**

Samples were washed once with 1× PBS and blocked with 2% BSA in PBS for 30 min at room temperature, labeling with blocking solution diluted antibodies was performed overnight at 4 °C, then the samples were washed 3 times with PBS and mounted with Prolong Gold with DAPI (Thermo Fisher). For optimized DNA staining, DAPI (Thermo Fisher) in PBS 10 min staining following manufacture’s manual was recommended especially when cells are dense, Prolong Gold without DAPI should be used for this option. HES1 Alexa 647 (1:200; Abcam), NR3C1 Alexa 488 (1:150; Cell signaling) and OCLN Alexa 594 (1:600; Thermo Fisher) are used for images shown.

**Oligo FISH of mRNA**

Stellaris™ predesigned human HES1 mRNA oligo FISH probe was bought from LGC Biosearch, samples are washed once with wash buffer (2× SSC, 10% formamide) and labeled with manufacturer’s protocol. Prolong Gold with DAPI could be used for easy handling and DAPI staining followed by Prolong Gold without DAPI mounting could be used for better DNA staining. OCLN antibody (EMD Millipore) and Alexa 488 secondary antibody (Thermo Fisher) are used in the pictures shown.

**3D confocal microscope imaging**

Nikon A-1 confocal microscope equipped with Diode based laser system for 405,488,561, and 640 excitations and operated with Nikon's Elements software in Microscopy & Image Analysis Laboratory of University of Michigan Medical School was used for this study. 1K×1K images were taken with 0.1 µM Z-stack (for the 60× objective lens) or with 0.2 µM Z-stack (for the 10× objective lens) steps covering all the signals to maximize details from the Z axis. 3D data sets are processed with Nikon's Elements software or the viewer on a 2K or 4K screen. Satisfying screenshots are clipped and shown.

**Data Records**

Caco2-BBe cells are seeded on flat and smooth hard glass coverclip surface and fixed at serial time points. We chose the fixation method compatible with DNA(BAC-FISH), RNA (Oligo-FISH) and immunofluorescence labelling to track DNA/RNA/protein forms of genes.
We chose a fixation method tested compatible for DNA(BAC-FISH)/RNA(Oligo-FISH)/Protein(IF) labelling and the samples can be stored for over 1 month for fixing the samples during the 21-day differentiation and labelled the Caco-2 cells with tight junction protein occludin and transcription factor HES1 and NR3C1. Cells are grown on the smooth hard glass surface and cannot form 3D spheres without 3D matrigel matrix support, we hypothesize that the actomyosin force driving the formation of 3D spheres generated the rotational patterns we have shown. We chose those interesting “geometrically informative” sight fields and recorded with confocal Z-stack images. This dataset may be useful to biophysicists, imaging data scientists, and mathematicians to test the applicability of “imaginary biological systems” to real biological systems following Alan Turing’s legacy guidance.

Imaging protocols, original and segmented data, and the source code are made publicly available on the project webpage: http://www.socr.umich.edu/projects/3d-cell-morphometry/data.html.

### Author contributions

G.Z. conceived the experiments shown in Fig. S1-S11.

A.K., I.D., W.M., S.Z. & J.W. participated discussion and editing.

All authors reviewed the manuscript.

### References

1. **Kalinin AA, Allyn-Feuer A, Ade A, Fon G-V, Meixner W, Dilworth D, de Wet JR, Higgins GA, Zheng G, Creekmore A, Wiley JW, Verdone JE, Veltri RW, Pienta KJ, Coffey DS, Athey BD, Dinov ID.** 3D cell nuclear morphology: microscopy imaging dataset and voxel-based morphometry classification results. *bioRxiv*. 2017; 208207.

2. **Rauzi M, Krzic U, Saunders TE, Krajnc M, Ziherl P, Hufnagel L, Leptin M.** Embryo-scale tissue mechanics during Drosophila gastrulation movements. *Nat Commun*. 2015; 6: 8677.

3. **Tashiro S, Lanctot C.** The International Nucleome Consortium. *Nucleus-Phila*. 2015; 6: 89-92.

4. **Rajapakse I, Smale S.** Emergence of function from coordinated cells in a tissue. *Proc Natl Acad Sci U S A*. 2017; 114: 1462-7.

5. **Zheng G, Victor Fon G, Meixner W, Creekmore A, Zong Y, M KD, Colacino J, Dedhia PH, Hong S, Wiley JW.** Chronic stress and intestinal barrier dysfunction: Glucocorticoid receptor and transcription repressor HES1 regulate tight junction protein Claudin-1 promoter. *Sci Rep*. 2017; 7: 4502.

6. **Rajapakse I, Smale S.** Mathematics of the Genome. *Found Comput Math*. 2017; 17: 1195-217.

7. **Gilmour D, Rembold M, Leptin M.** From morphogen to morphogenesis and back. *Nature*. 2017; 541: 311-20.

8. **Uhler C, Shivashankar GV.** Regulation of genome organization and gene expression by nuclear mechanotransduction. *Nat Rev Mol Cell Biol*. 2017; 18: 717-27.

9. **Ronquist S, Patterson G, Muir LA, Lindsly S, Chen H, Brown M, Wicha MS, Bloch A, Brockett R, Rajapakse I.** Algorithm for cellular reprogramming. *Proc Natl Acad Sci U S A*. 2017; 114: 11832-7.

10. **Turing AM.** The Chemical Basis of Morphogenesis. *Philos T Roy Soc B*. 1952; 237: 37-72.

11. **Ball P.** Forging patterns and making waves from biology to geology: a commentary on Turing (1952) 'The chemical basis of morphogenesis'. *Philos Trans R Soc Lond B Biol Sci*. 2015; 370.

12. **Kumar A, Maitra A, Sumit M, Ramaswamy S, Shivashankar GV.** Actomyosin contractility rotates the cell nucleus. *Sci Rep-Uk*. 2014; 4.

13. **Camley BA, Zhang Y, Zhao Y, Li B, Ben-Jacob E, Levine H, Rappel WJ.** Polarity mechanisms such as contact inhibition of locomotion regulate persistent rotational motion of mammalian cells on micropatterns. *Proc Natl Acad Sci U S A*. 2014; 111: 14770-5.

14. **Wang H, Lacoche S, Huang L, Xue B, Muthuswamy SK.** Rotational motion during three-dimensional morphogenesis of mammary epithelial acini relates to laminin matrix assembly. *Proc Natl Acad Sci U S A*. 2013; 110: 163-8.

15. **Murrell M, Oakes PW, Lenz M, Gardel ML.** Forcing cells into shape: the mechanics of actomyosin contractility. *Nat Rev Mol Cell Biol*. 2015; 16: 486-98.

16. **Lozoya OA, Gilchrist CL, Guilak F.** Universally Conserved Relationships between Nuclear Shape and Cytoplasmic Mechanical Properties in Human Stem Cells. *Sci Rep*. 2016; 6: 23047.

17. **Alam SG, Zhang Q, Prasad N, Li Y, Chamala S, Kuchibhotla R, Kc B, Aggarwal V, Shrestha S, Jones AL, Levy SE, Roux KJ, Nickerson JA, Lele TP.** The mammalian LINC complex regulates genome transcriptional responses to substrate rigidity. *Sci Rep*. 2016; 6: 38063.

18. **Le HQ, Ghatak S, Yeung CY, Tellkamp F, Gunschmann C, Dieterich C, Yeroslaviz A, Habermann B, Pombo A, Niessen CM, Wickstrom SA.** Mechanical regulation of transcription controls Polycomb-mediated gene silencing during lineage commitment. *Nat Cell Biol*. 2016; 18: 864-75.

19. **Cho S, Irianto J, Discher DE.** Mechanosensing by the nucleus: From pathways to scaling relationships. *J Cell Biol*. 2017; 216: 305-15.

20. **Karalyan ZA, Djaghatspanyan NG, Gasparyan MH, Hakobyan LA, Abroyan LO, Magakyan YH, Ter-Pogossyan ZR, Kamalyan LA, Karalova EM.** Morphometry of nuclear and nucleolar structures in a CaCo-2 cell line. *Cell Biol Int*. 2004; 28: 249-53.

21. **Hernandez M, Patzig J, Mayoral SR, Costa KD, Chan JR, Casaccia P.** Mechanostimulation Promotes Nuclear and Epigenetic Changes in Oligodendrocytes. *J Neurosci*. 2016; 36: 806-13.

22. **Tan Y, Tajik A, Chen J, Jia Q, Chowdhury F, Wang L, Chen J, Zhang S, Hong Y, Yi H, Wu DC, Zhang Y, Wei F, Poh YC, Seong J, Singh R, Lin LJ, Doganay S, Li Y, Jia H, Ha T, Wang Y, Huang B, Wang N.** Matrix softness regulates plasticity of tumour-repopulating cells via H3K9 demethylation and Sox2 expression. *Nat Commun*. 2014; 5: 4619.

23. **Arnold TR, Stephenson RE, Miller AL.** Rho GTPases and actomyosin: Partners in regulating epithelial cell-cell junction structure and function. *Exp Cell Res*. 2017; 358: 20-30.

24. **Quiros M, Nusrat A.** RhoGTPases, actomyosin signaling and regulation of the epithelial Apical Junctional Complex. *Semin Cell Dev Biol*. 2014; 36: 194-203.

25. **Zihni C, Balda MS, Matter K.** Signalling at tight junctions during epithelial differentiation and microbial pathogenesis. *J Cell Sci*. 2014; 127: 3401-13.

26. **Ivanov AI, Hopkins AM, Brown GT, Gerner-Smidt K, Babbin BA, Parkos CA, Nusrat A.** Myosin II regulates the shape of three-dimensional intestinal epithelial cysts. *J Cell Sci*. 2008; 121: 1803-14.

27. **Trietsch SJ, Naumovska E, Kurek D, Setyawati MC, Vormann MK, Wilschut KJ, Lanz HL, Nicolas A, Ng CP, Joore J, Kustermann S, Roth A, Hankemeier T, Moisan A, Vulto P.** Membrane-free culture and real-time barrier integrity assessment of perfused intestinal epithelium tubes. *Nat Commun*. 2017; 8: 262.

28. **Suzuki T.** Regulation of intestinal epithelial permeability by tight junctions. *Cell Mol Life Sci*. 2013; 70: 631-59.

29. **Moore T, Wu SK, Michael M, Yap AS, Gomez GA, Neufeld Z.** Self-organizing actomyosin patterns on the cell cortex at epithelial cell-cell junctions. *Biophys J*. 2014; 107: 2652-61.

30. **Gorfinkiel N.** From actomyosin oscillations to tissue-level deformations. *Dev Dyn*. 2016; 245: 268-75.

31. **Carminati M, Gallini S, Pirovano L, Alfieri A, Bisi S, Mapelli M.** Concomitant binding of Afadin to LGN and F-actin directs planar spindle orientation. *Nat Struct Mol Biol*. 2016; 23: 155-63.

32. **Durgan J, Kaji N, Jin D, Hall A.** Par6B and atypical PKC regulate mitotic spindle orientation during epithelial morphogenesis. *J Biol Chem*. 2011; 286: 12461-74.

33. **Jaffe AB, Kaji N, Durgan J, Hall A.** Cdc42 controls spindle orientation to position the apical surface during epithelial morphogenesis. *J Cell Biol*. 2008; 183: 625-33.

34. **Iwao T, Toyota M, Miyagawa Y, Okita H, Kiyokawa N, Akutsu H, Umezawa A, Nagata K, Matsunaga T.** Differentiation of human induced pluripotent stem cells into functional enterocyte-like cells using a simple method. *Drug Metab Pharmacokinet*. 2014; 29: 44-51.

35. **Lepage M, Seltana A, Thibault MP, Tremblay E, Beaulieu JF.** Knockdown of laminin alpha5 stimulates intestinal cell differentiation. *Biochem Biophys Res Commun*. 2018; 495: 1510-5.

36. **Odenwald MA, Choi W, Buckley A, Shashikanth N, Joseph NE, Wang Y, Warren MH, Buschmann MM, Pavlyuk R, Hildebrand J, Margolis B, Fanning AS, Turner JR.** ZO-1 interactions with F-actin and occludin direct epithelial polarization and single lumen specification in 3D culture. *J Cell Sci*. 2017; 130: 243-59.

37. **Dionne LK, Wang XJ, Prekeris R.** Midbody: from cellular junk to regulator of cell polarity and cell fate. *Curr Opin Cell Biol*. 2015; 35: 51-8.

38. **Brangwynne C, Huang S, Parker KK, Ingber DE, Ostuni E.** Symmetry breaking in cultured mammalian cells. *In Vitro Cell Dev Biol Anim*. 2000; 36: 563-5.

39. **Seo D, Southard KM, Kim JW, Lee HJ, Farlow J, Lee JU, Litt DB, Haas T, Alivisatos AP, Cheon J, Gartner ZJ, Jun YW.** A Mechanogenetic Toolkit for Interrogating Cell Signaling in Space and Time. *Cell*. 2016; 165: 1507-18.

40. **Gordon WR, Zimmerman B, He L, Miles LJ, Huang J, Tiyanont K, McArthur DG, Aster JC, Perrimon N, Loparo JJ, Blacklow SC.** Mechanical Allostery: Evidence for a Force Requirement in the Proteolytic Activation of Notch. *Dev Cell*. 2015; 33: 729-36.

41. **LaFoya B, Munroe JA, Mia MM, Detweiler MA, Crow JJ, Wood T, Roth S, Sharma B, Albig AR.** Notch: A multi-functional integrating system of microenvironmental signals. *Dev Biol*. 2016; 418: 227-41.

42. **Chowdhury F, Li IT, Ngo TT, Leslie BJ, Kim BC, Sokoloski JE, Weiland E, Wang X, Chemla YR, Lohman TM, Ha T.** Defining Single Molecular Forces Required for Notch Activation Using Nano Yoyo. *Nano Lett*. 2016; 16: 3892-7.

43. **Kovall RA, Gebelein B, Sprinzak D, Kopan R.** The Canonical Notch Signaling Pathway: Structural and Biochemical Insights into Shape, Sugar, and Force. *Dev Cell*. 2017; 41: 228-41.

44. **Real PJ, Tosello V, Palomero T, Castillo M, Hernando E, de Stanchina E, Sulis ML, Barnes K, Sawai C, Homminga I, Meijerink J, Aifantis I, Basso G, Cordon-Cardo C, Ai W, Ferrando A.** Gamma-secretase inhibitors reverse glucocorticoid resistance in T cell acute lymphoblastic leukemia. *Nat Med*. 2009; 15: 50-8.

45. **Lemke U, Krones-Herzig A, Berriel Diaz M, Narvekar P, Ziegler A, Vegiopoulos A, Cato AC, Bohl S, Klingmuller U, Screaton RA, Muller-Decker K, Kersten S, Herzig S.** The glucocorticoid receptor controls hepatic dyslipidemia through Hes1. *Cell Metab*. 2008; 8: 212-23.

46. **Revollo JR, Oakley RH, Lu NZ, Kadmiel M, Gandhavadi M, Cidlowski JA.** HES1 is a master regulator of glucocorticoid receptor-dependent gene expression. *Sci Signal*. 2013; 6: ra103.

47. **Dunn SJ, Appleton PL, Nelson SA, Nathke IS, Gavaghan DJ, Osborne JM.** A two-dimensional model of the colonic crypt accounting for the role of the basement membrane and pericryptal fibroblast sheath. *PLoS Comput Biol*. 2012; 8: e1002515.

48. **Benoit YD, Lepage MB, Khalfaoui T, Tremblay E, Basora N, Carrier JC, Gudas LJ, Beaulieu JF.** Polycomb repressive complex 2 impedes intestinal cell terminal differentiation. *J Cell Sci*. 2012; 125: 3454-63.

49. **Phillips NE, Manning CS, Pettini T, Biga V, Marinopoulou E, Stanley P, Boyd J, Bagnall J, Paszek P, Spiller DG, White MR, Goodfellow M, Galla T, Rattray M, Papalopulu N.** Stochasticity in the miR-9/Hes1 oscillatory network can account for clonal heterogeneity in the timing of differentiation. *Elife*. 2016; 5.

50. **Torii KU.** Two-dimensional spatial patterning in developmental systems. *Trends Cell Biol*. 2012; 22: 438-46.

51. **Toth B, Ben-Moshe S, Gavish A, Barkai N, Itzkovitz S.** Early commitment and robust differentiation in colonic crypts. *Mol Syst Biol*. 2017; 13: 902.

52. **Kay SK, Harrington HA, Shepherd S, Brennan K, Dale T, Osborne JM, Gavaghan DJ, Byrne HM.** The role of the Hes1 crosstalk hub in Notch-Wnt interactions of the intestinal crypt. *PLoS Comput Biol*. 2017; 13: e1005400.

53. **Guo XK, Ou J, Liang S, Zhou X, Hu X.** Epithelial Hes1 maintains gut homeostasis by preventing microbial dysbiosis. *Mucosal Immunol*. 2018.

54. **Riahi R, Sun J, Wang S, Long M, Zhang DD, Wong PK.** Notch1-Dll4 signalling and mechanical force regulate leader cell formation during collective cell migration. *Nat Commun*. 2015; 6: 6556.

55. **Meloty-Kapella L, Shergill B, Kuon J, Botvinick E, Weinmaster G.** Notch ligand endocytosis generates mechanical pulling force dependent on dynamin, epsins, and actin. *Dev Cell*. 2012; 22: 1299-312.

56. **Chen H, Chen J, Muir LA, Ronquist S, Meixner W, Ljungman M, Ried T, Smale S, Rajapakse I.** Functional organization of the human 4D Nucleome. *Proc Natl Acad Sci U S A*. 2015; 112: 8002-7.

57. **Moor AE, Golan M, Massasa EE, Lemze D, Weizman T, Shenhav R, Baydatch S, Mizrahi O, Winkler R, Golani O, Stern-Ginossar N, Itzkovitz S.** Global mRNA polarization regulates translation efficiency in the intestinal epithelium. *Science*. 2017; 357: 1299-303.

58. **Dey-Guha I, Wolfer A, Yeh AC, J GA, Darp R, Leon E, Wulfkuhle J, Petricoin EF, 3rd, Wittner BS, Ramaswamy S.** Asymmetric cancer cell division regulated by AKT. *Proc Natl Acad Sci U S A*. 2011; 108: 12845-50.

59. **Weiland Y, Lemmer P, Cremer C.** Combining FISH with localisation microscopy: Super-resolution imaging of nuclear genome nanostructures. *Chromosome Res*. 2011; 19: 5-23.

**Figures**


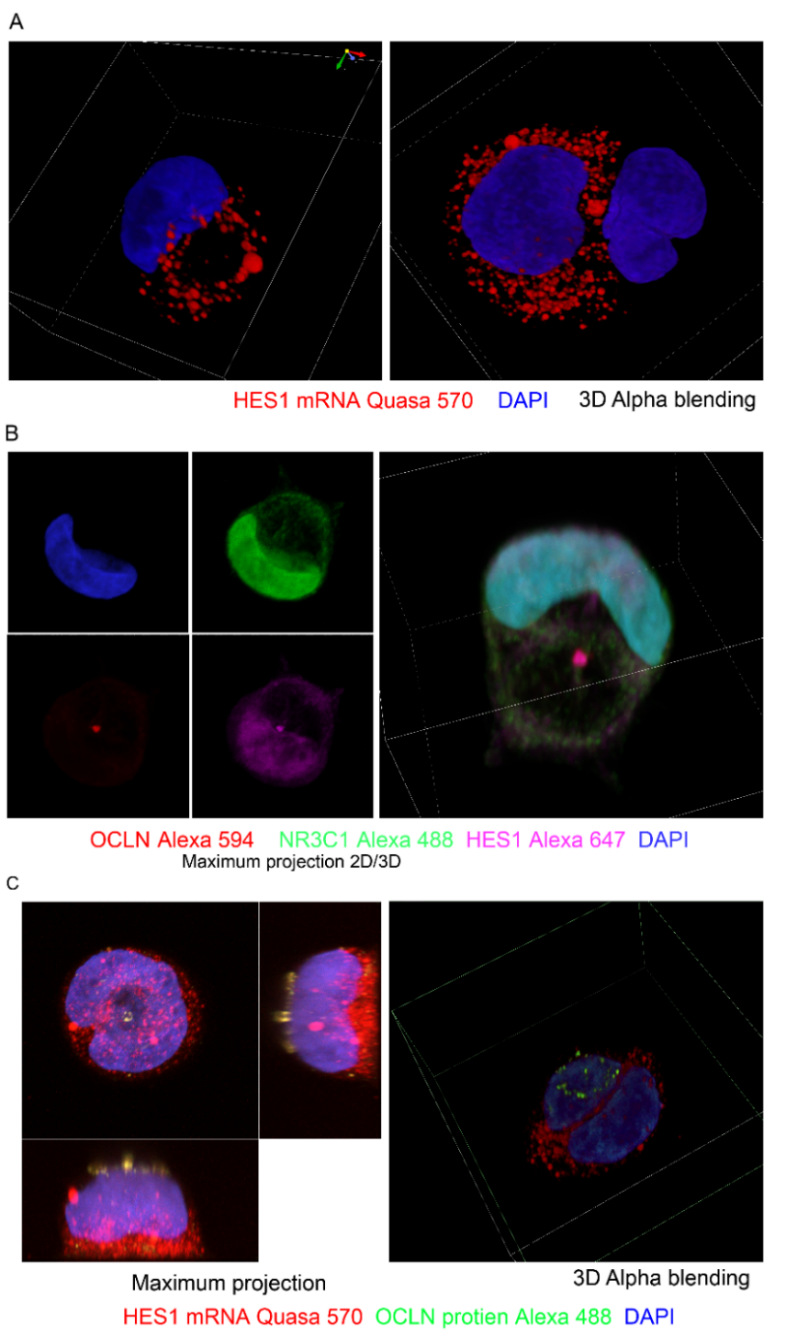


**Fig. S1. Ring patterns in 1-2 Caco-2 BBe cells at day 2**

1. HES1 mRNA showed circular distribution in 1 cell and neighboring cells with or without HES1 mRNA are observed.
2. Caco-2 BBe cells on day 1 cover clip are labelled with OCLN, NR3C1 and HES1 antibodies. Single cell genome with OCLN HES1 morphogen center.
3. Caco-2 BBe cells on day 1 cover clip are labelled with HES1 mRNA Qausa 570 oligo FISH probes and OCLN antibody. Single cell genome labelled with DAPI can be found in a torus shape. OCLN protein showed circular wave pattern, this pattern may correlate with actomyosin force [29]. Formation of “ring of cells” proposed by Turing may start from 1 to 2 cells, the HES1 distribution may conform to “The morphogen pattern in a ring of cells as deduced by Turing” [11].





**Fig. S2. 2 cells spreading spirally on flat 2D flat surface.**

2 Caco-2 BBe cells on day 3 cover clip labelled with OCLN, NR3C1, and HES1 antibodies. Spirally spreading OLCN and HES1 can be seen. NR3C1 showed wave peak pattern comes from the spiral axis. OCLN’s circular wave pattern may correlate with actomyosin force [29]. The shape of the nucleuses looks conform to actomyosin Rho GTPase shape in a model of the rotational motion of polarized mammalian epithelial cells on micropatterns [13].


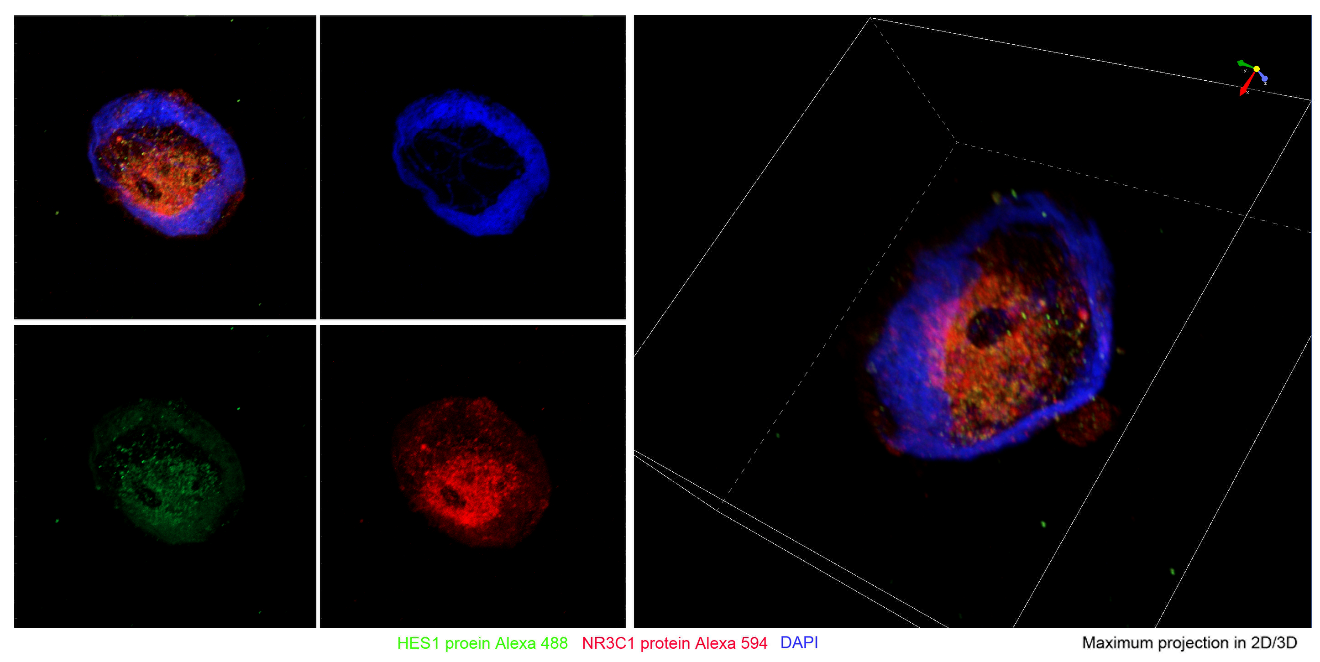


**Fig. S3. Vortex/Horn shaped cell with ring shaped genome.**

Caco-2 BBe cells on day 1 cover clip are labelled with NR3C1 and HES1 antibodies. DNA staining showed circular loop pattern, a hole can be seen located at the center of a vortex. Diffusion of the transcription factor NR3C1 and HES1 from the hole can be seen. This pattern may correlate with actomyosin force [29]. The shape of genome may conform to a “Hopf bifurcation” used to illustrate the mathematics of the genome [6].


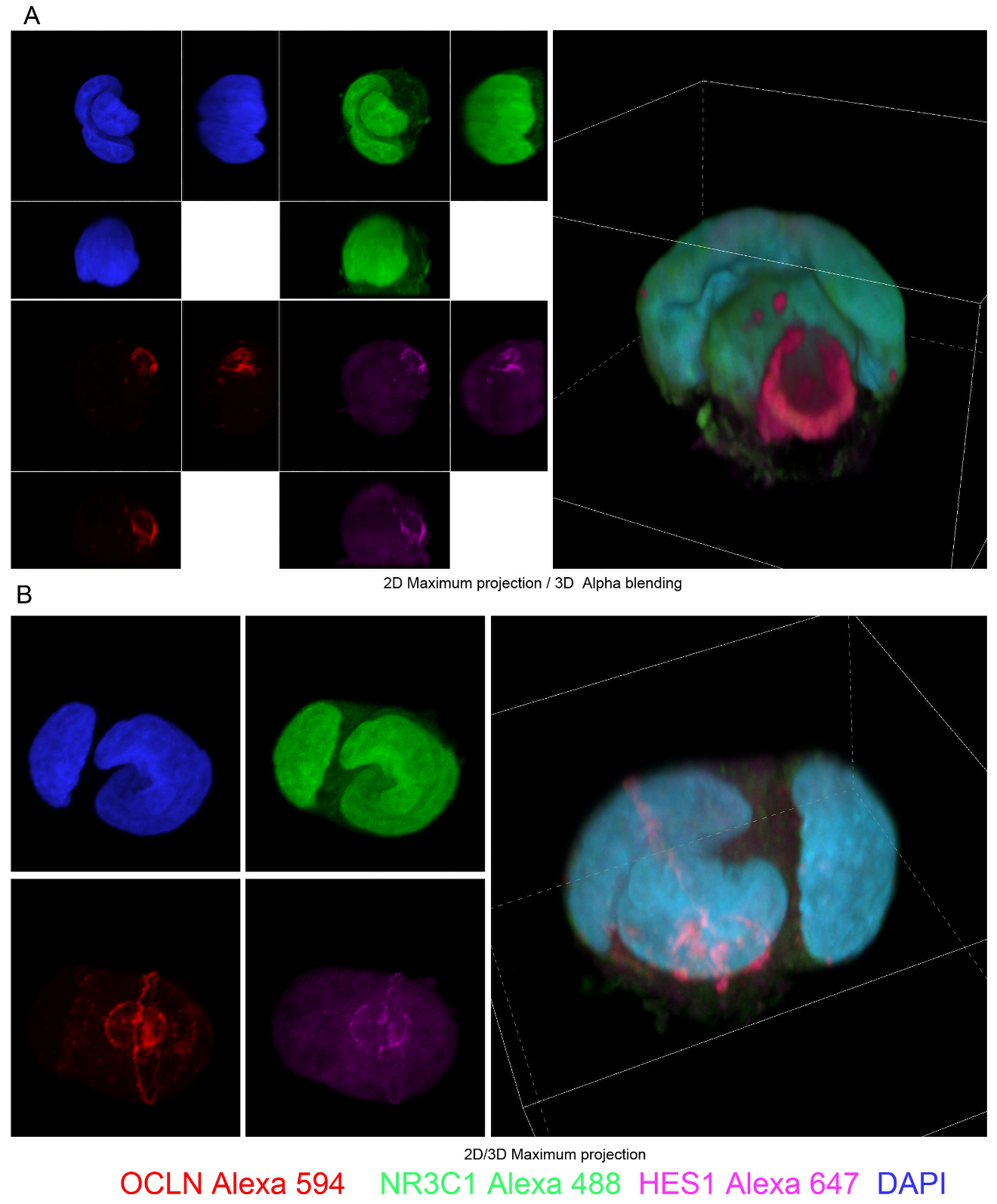


**Fig. S4. OCLN rings on sphere surface of the cells.**

Caco-2 BBe cells on day 1 cover clips are labelled with OCLN, NR3C1, and HES1 antibodies. OCLN showed ring patterns indicating different eccentricity values (circle/parabola/hyperbola) on the sphere surface of non-separated nucleuses.


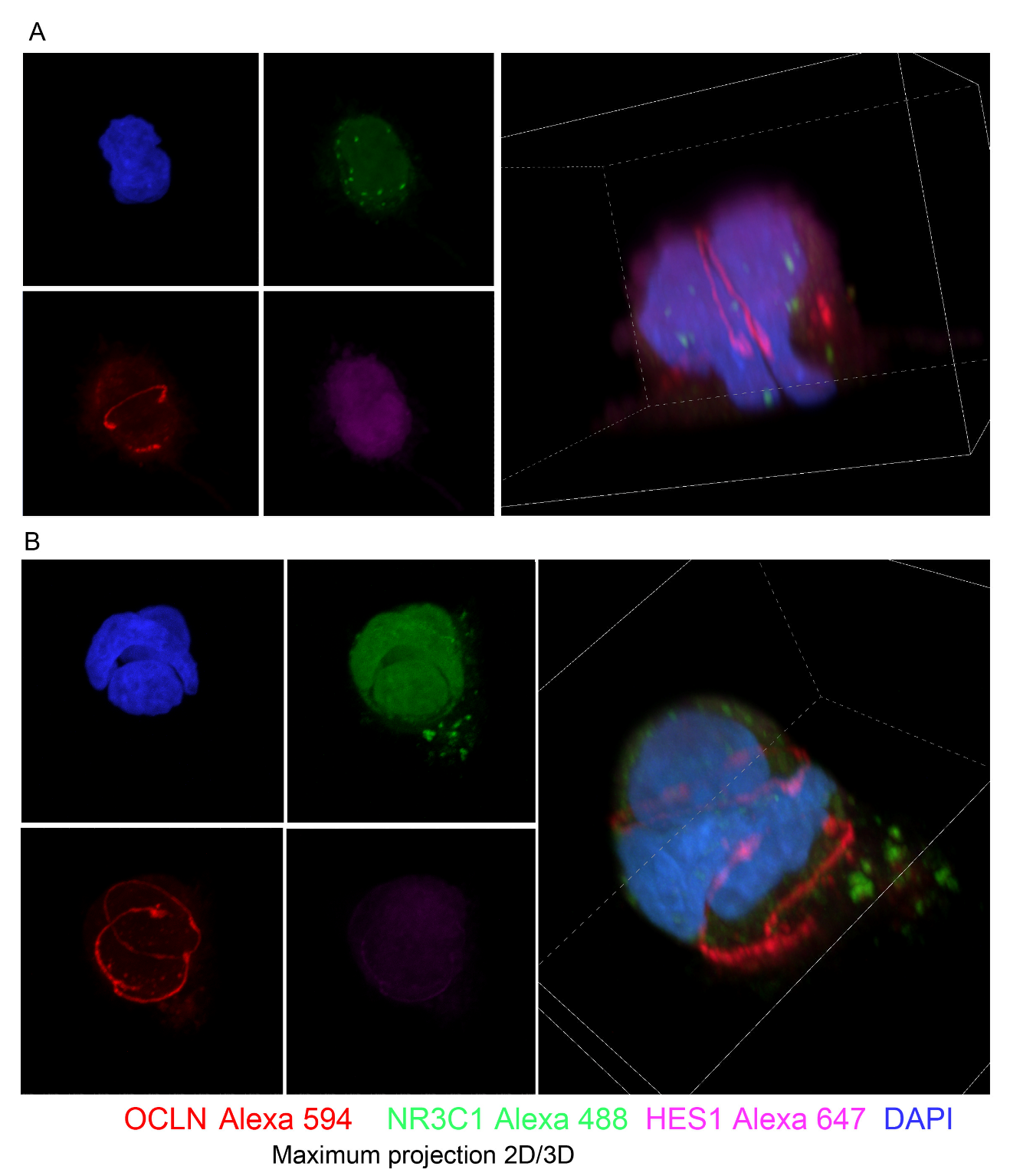


**Fig. S5. OCLN orbit tracks between separating nucleuses**

Caco-2 BBe cells on day 1 (A) / day 3 (B) cover clips are labelled with OCLN, NR3C1, and HES1 antibodies. OCLN track showed bright ellipse orbits like pattern.


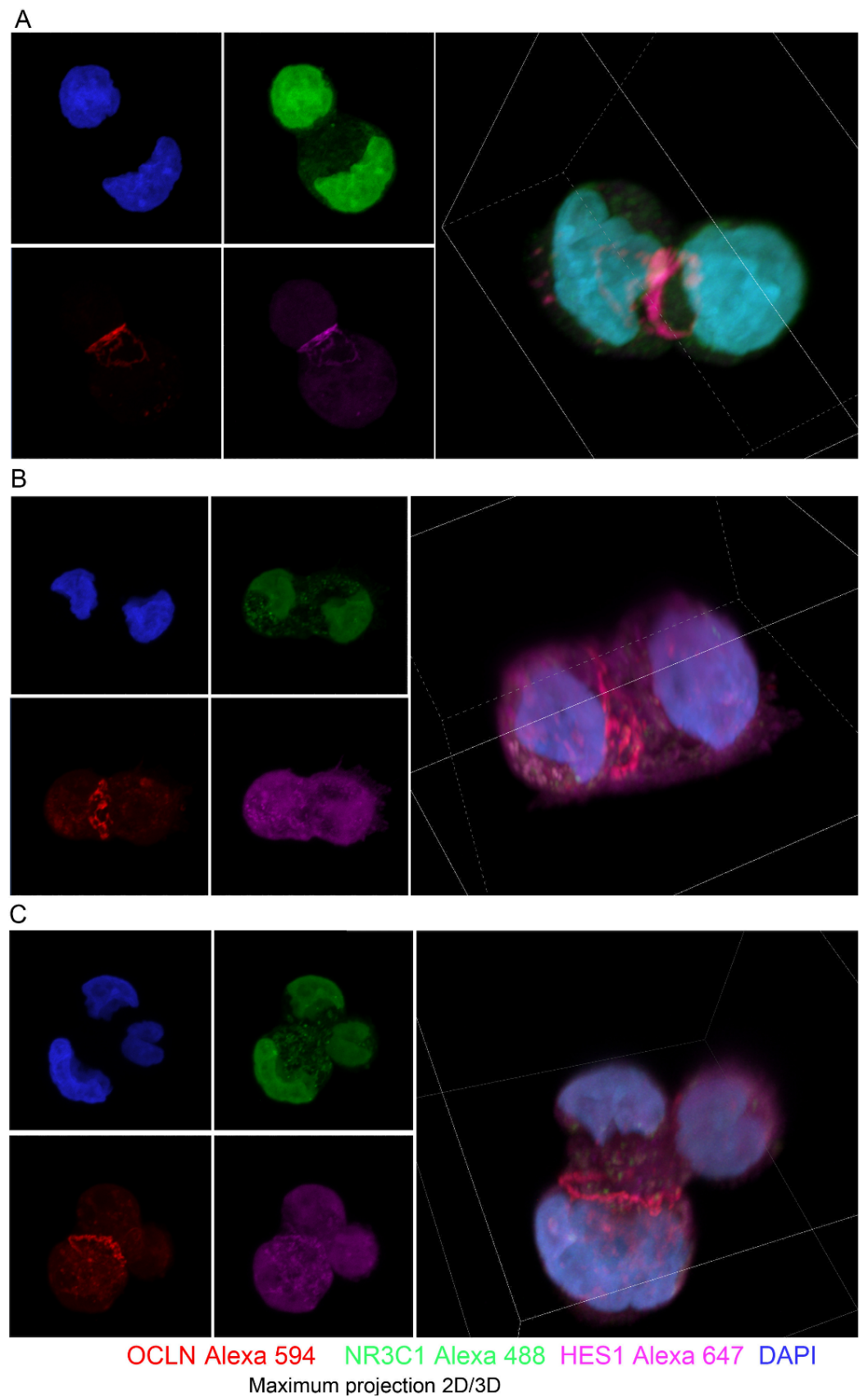


**Fig. S6. OCLN contractile ring between separated nucleuses**

Caco-2 BBe cells on day 1 cover clips are labelled with OCLN, NR3C1, and HES1 antibodies. OCLN showed contractile ring pattern separating the cells with separated nucleuses. The shape of the nucleuses looks conform to actomyosin Rho GTPase shape in a model of the rotational motion of polarized mammalian epithelial cells on micropatterns [13].


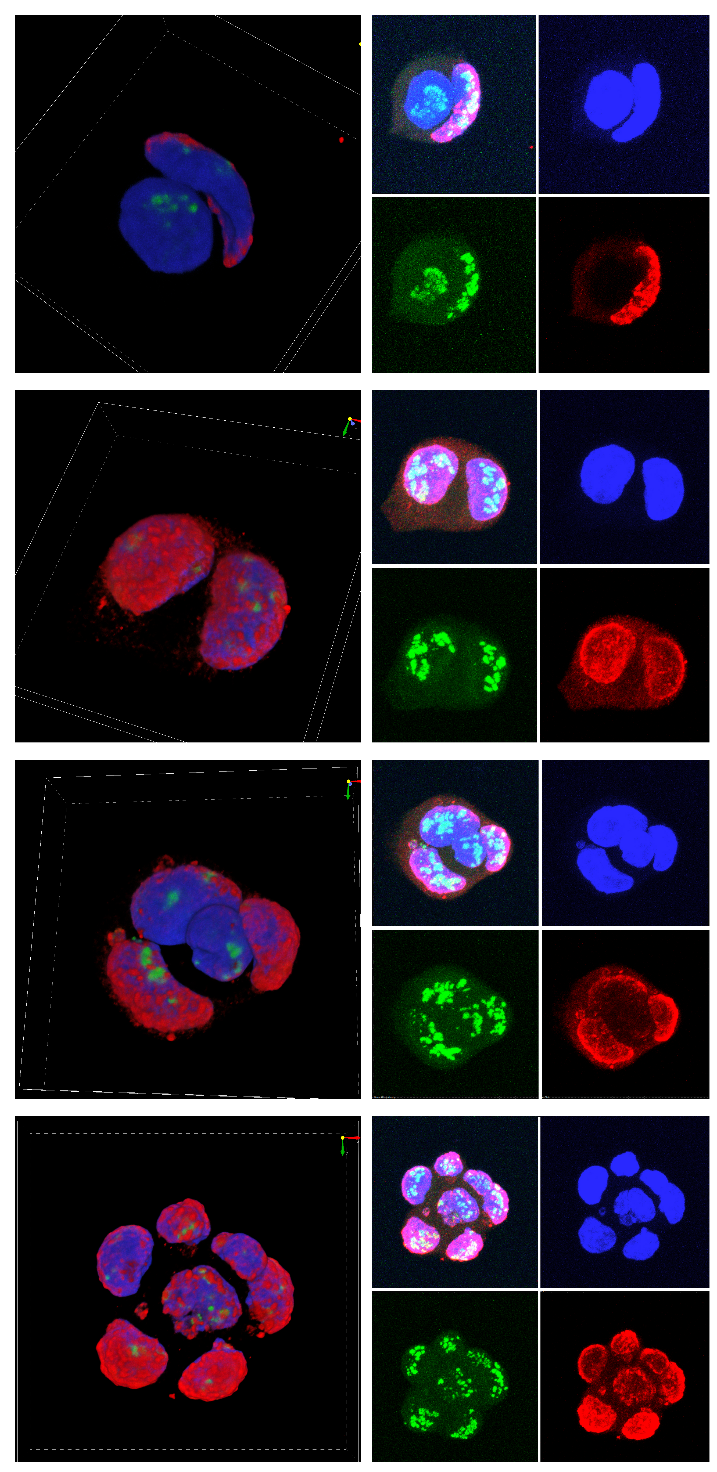


**Fig. S7. H3K9me3 & nucleoli in nucleuses**

Caco-2 BBe cells on day 1 / day3 cover clips are labelled with H3K9me3 and fibrillin antibodies. Rotational patterns of H3K9me3 can be seen, there might be a correlation of H3K9me3 with mechanic forces [21].


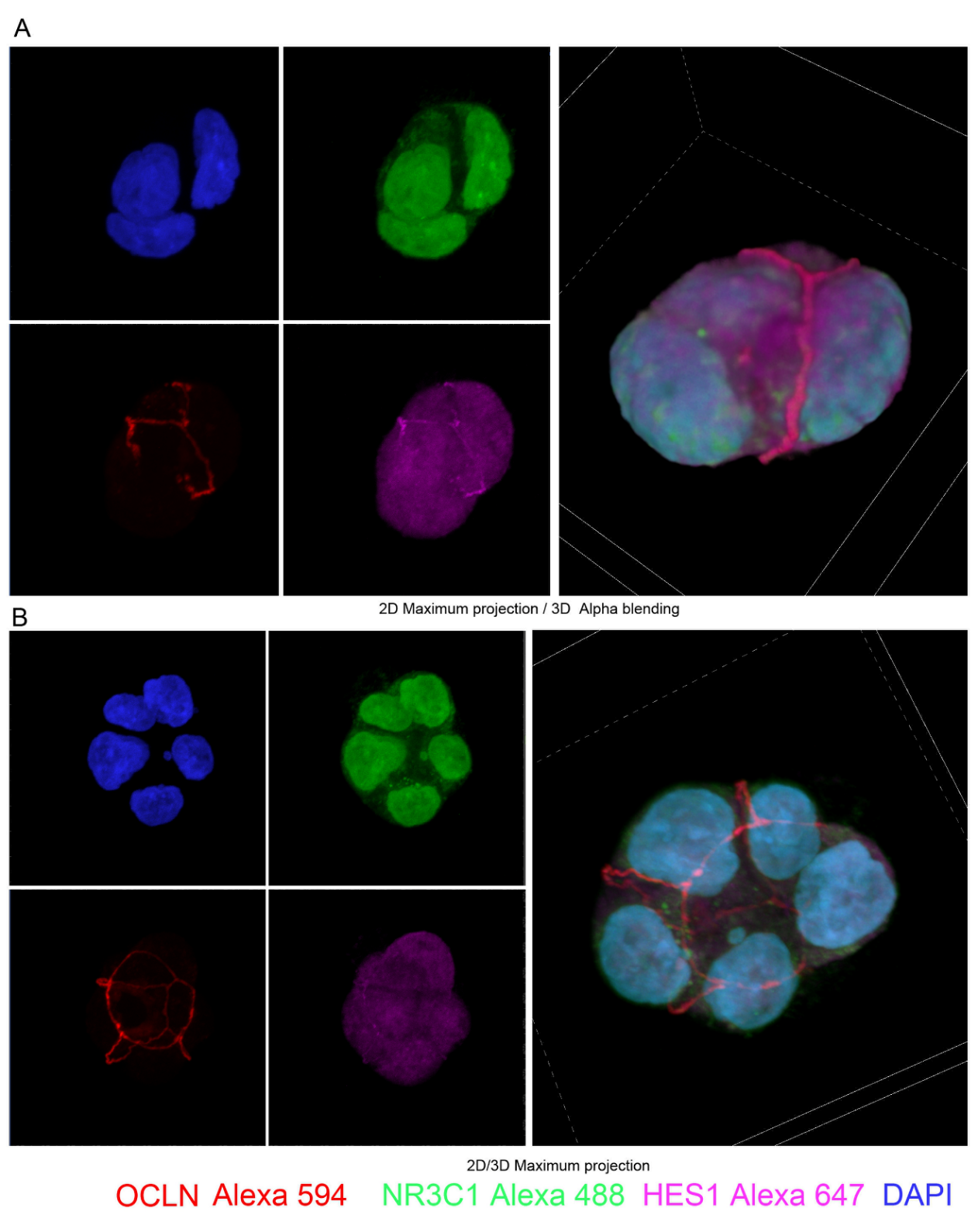


**Fig. S8. Tendency of forming 3D structures.**

Caco-2 BBe cells on day 1 cover clips are labelled with OCLN, NR3C1, and HES1 antibodies. Cells showed 3D organization, OCLN showed 3D orbit tracks like pattern. This pattern may correlate with rotational motion observed during 3D morphogenesis of epithelial cell morphogenesis [14]. Euler characteristic should be considered.


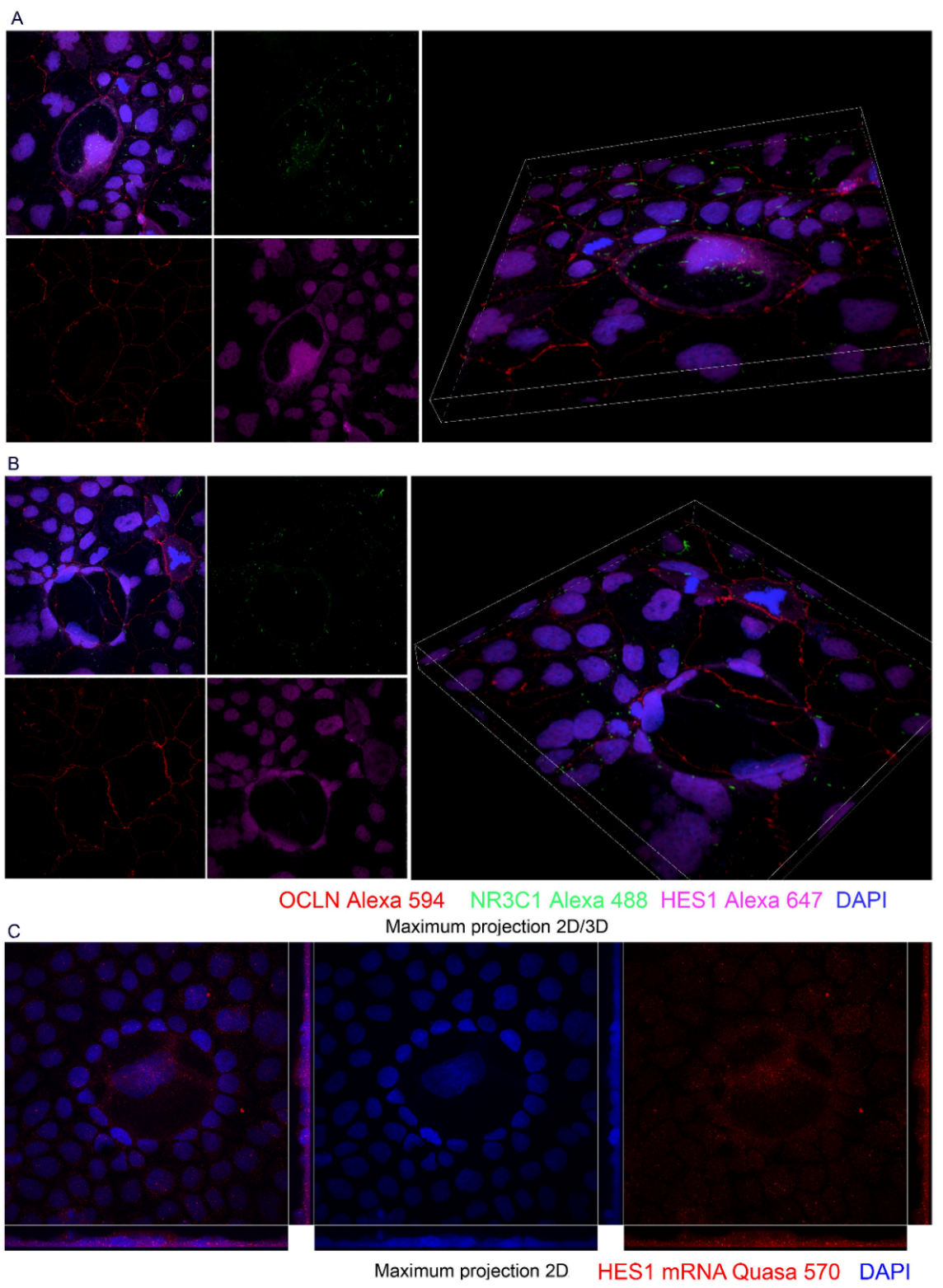


**Fig. S9. Circular spreading cells on flat 2D surface.**

Caco-2 BBe cells on day 4 cover clips are labelled with OCLN, NR3C1, and HES1 antibodies. Formation of “ring of cells” may conform to “The morphogen pattern in a ring of cells as deduced by Turing” when HES1 is considered as the morphogen [11].

1. Spirally spreading HES1 wave toward OCLN ring from the nucleus.
2. HES1 protein ring guided ring of cells.
3. HES1 mRNA ring guided ring of cells, HES1 mRNA showed bridge like pattern within the ring.


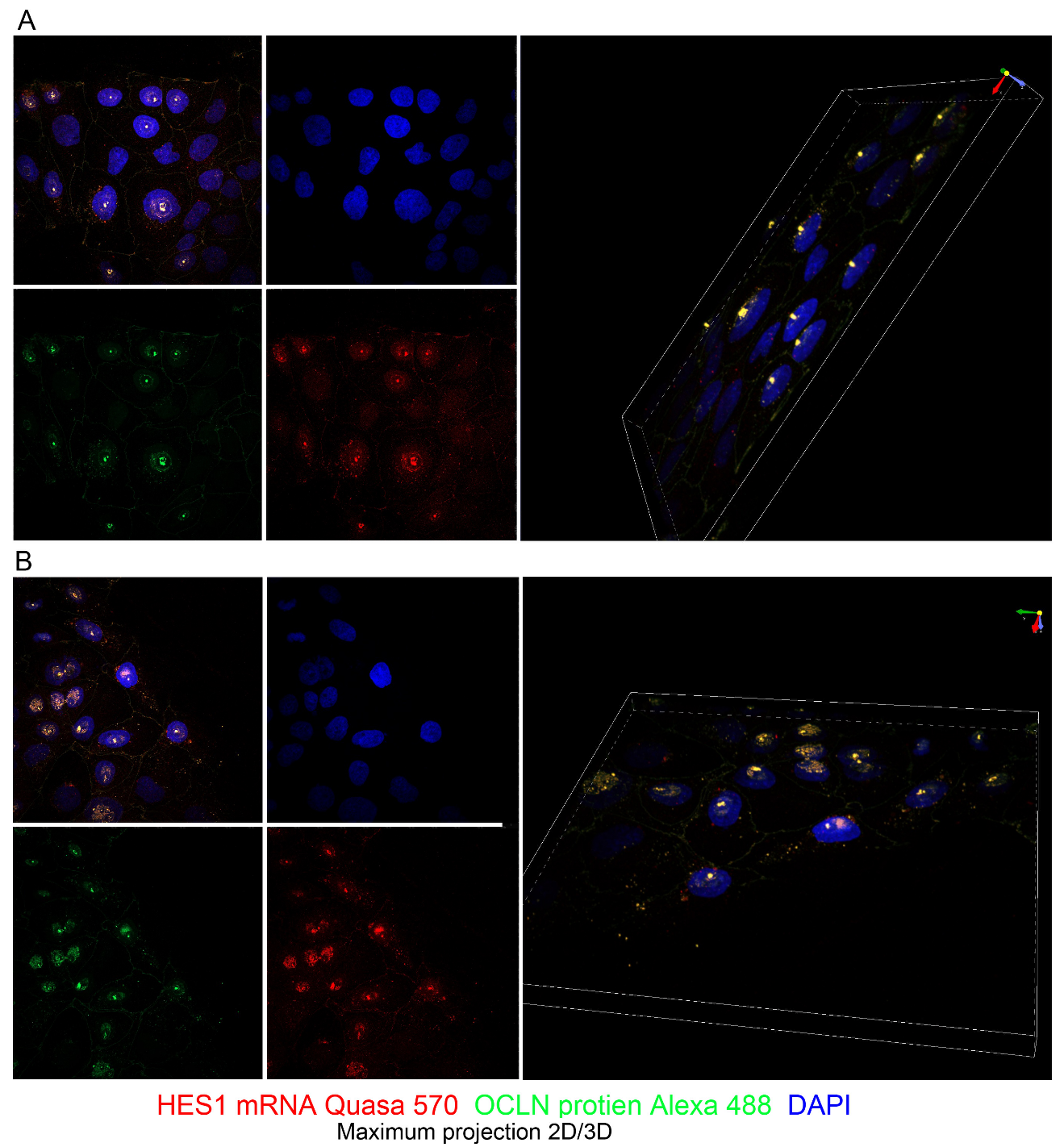


**Fig. S10. Turing pattern of cells with or without HES1 mRNA / OCLN protein spirally spreading from pole of cell polarization like circular waves.**

Caco-2 BBe cells on day 4 cover clips are labelled with HES1 mRNA and OCLN protein. HES1 on and off pattern is similar to what was observed in colon crypts which conform to Turing patterning [51]. Circular wave shaped HES1 mRNA and OCLN protein can be observed.


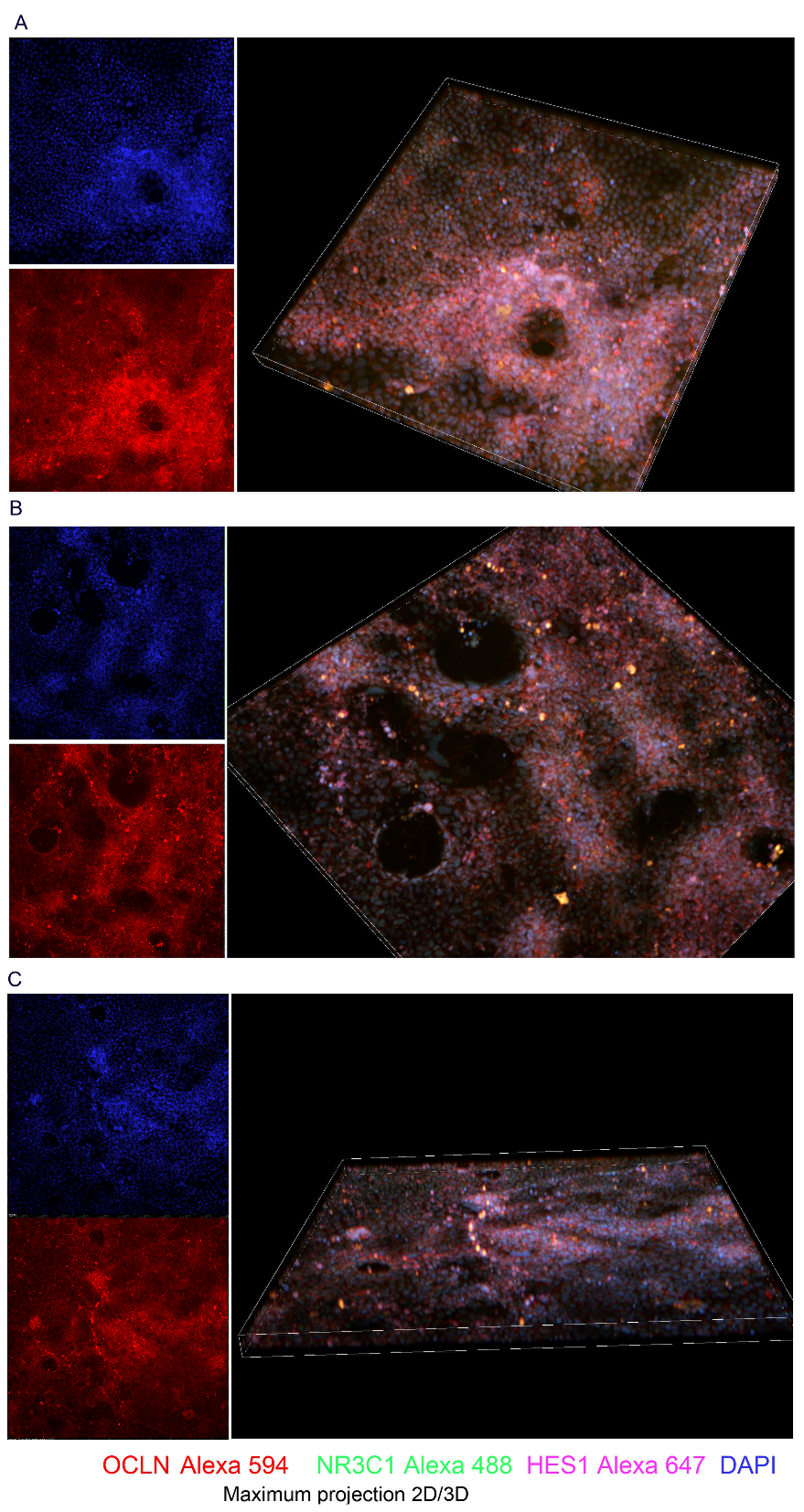


**Fig. S11. Huge rings of cells and circular waves of cells.**

A. Caco-2 BBe cells on day 21 were treated with vehicle for 2 h, cover clips are labelled with OCLN, NR3C1, and HES1 antibodies.

B, C. Caco-2 BBe cells on day 20 were treated with 500 nM GC for 24 h, cover clips are labelled with OCLN, NR3C1, and HES1 antibodies.
